# Arthroscopic pie-crusting release of the posteromedial complex of the knee for surgical treatment of medial meniscus injury

**DOI:** 10.1186/s12891-020-03336-9

**Published:** 2020-05-14

**Authors:** Xu Han, Peizhao Wang, Jinyang Yu, Xiao Wang, Honglue Tan

**Affiliations:** 1 Henan Luoyang Orthopedic-Traumatological Hospital (Henan Orthopedic Hospital), No.80 Qingming southern Road, Luoyang, 471002 China; 2 Hunan University of Chinese Traditional Medicine, Changsa, 410208 Hunan China

**Keywords:** Posteromedial complex, Meniscus injury, Pie-crusting release, Arthroscopy, Knee

## Abstract

**Background:**

An arthroscopic narrow posteromedial gap of the knee may cause failure of a meniscus operation. The posteromedial complex (PMC) of the knee, including the posterior part of the medial collateral ligament (MCL) and the posterior oblique ligament (POL), has a restrictive effect on the opening of the posteromedial gap of the knee in the half-extension position. Thus, we evaluated the radiological and clinical results of pie-crusting release of the PMC for arthroscopic meniscal surgery in tight knees.

**Methods:**

Sixty patients with posterior injury of the medial meniscus were reviewed. All patients accepted arthroscopic pie-crusting release of the PMC. Fourty patients accepted meniscoplasty, and 20 patients accepted meniscal suturing. To evaluate the arthroscopic opening of the medial gap in 20° half-extension under 11-kg valgus stress, the width of the medial space before and after release were measured. During follow-up, the medial stability was evaluated by radiographic measurements of the joint space width (JSW) in 20° half-extension. Magnetic resonance imaging (MRI) was conducted to evaluate healing of the MCL and meniscus. Knee functions were evaluated using VAS (visual analogy score), Lysholm, IKDC (International Knee Documentation Committee) and Tegner scoring systems.

**Results:**

In all patients, meniscus operations were performed without iatrogenic cartilage injury. After PMC release, the arthroscopic width of the medial space was 5.7 ± 0.5 mm, larger than that before release (2.5 ± 0.5 mm, *p* < 0.01). The follow-up time was 21.93 ± 7.04 months, there was no residual valgus laxity of the knee. The radiographic JSW was 5.97 ± 0.8 mm preoperatively, 9.2 ± 1.1 mm in the 1st week postoperatively, and 6.1 ± 0.9 mm by the 3rd postoperative month, showing no differences between preoperative and 3 months postoperative measurement (*p* > 0.05). For sutured meniscus, MRI showed healing in 15 patients while five had two-grade abnormal signals. VAS, Lysholm, IKDC and Tegner scores were 1.80 ± 0.51, 80.08 ± 3.74, 82.17 ± 4.64 and 5.48 ± 0.59, respectively, showing significant differences compared with the preoperative scores (5.57 ± 0.69, 48.17 ± 4.22, 51.42 ± 4.02 and 3.20 ± 0.68, respectively, *p*< 0.01).

**Conclusions:**

Pie-crusting release of the PMC can increase the posteromedial space and improve the visual field of the knee under arthroscopy, while neither causing no residual valgus instability of the knee nor affecting the clinical outcome at the final follow-up.

## Background

Medial meniscus injury is a common cause of knee joint pain. If it is not properly treated in a timely manner, degenerative changes of the cartilage in the medial compartment of the knee joint will happen [1]. With the rapid development of arthroscopic minimally-invasive technology, this kind of injury involving the posterior part of the medial meniscus can be treated by means of microscopic repair or suture according to the different injury types, which ultimately avoids the occurrence or development of osteoarthritis [2]. However, during arthroscopic surgery, an unclear visual field of the posterior meniscus caused by the narrow medial space of the knee joint is often encountered, which commonly leads to inaccurate surgical instrument operation, iatrogenic cartilage injury, excessive cutting of the meniscus and the failure of meniscus suture [3]. To address these problems, recent publications have reported local release of MCL through different methods to increase the gap of the posteromedial compartment, thereby improving the surgical visual field and the operating space under microscopy, and achieving satisfactory clinical results of medial meniscus surgery [2–6]. However, in these studies, the authors suggested MCL release without showing the specific release location, and some authors recommend that MCL release be performed with the assistance of a medial incision.

It is well known that the medial restrictive structure of the knee joint includes not only the MCL, but also the posterior oblique ligament (POL) and the capsule, collectively termed the posteromedial complex (PMC); different components of the PMC play different roles in knee flexion and extension [7]. During arthroscopic surgery of the posterior part of the medial meniscus with the knee joint in the half-extension position, the medial and posterior structures of the knee exert a restrictive effect on enlargement of the posteromedial space. Therefore, the structures being released should include the posterior part of the MCL, the POL and capsule. In this study, percutaneous pie-crusting release was performed at the medial and posterior part of the knee joint under valgus stress in the knee half-extension position, and the effect of release on the visual field of the posteromedial space of the knee joint was evaluated. The postoperative effect of release on the medial stability of the knee and the clinical efficacy of meniscal surgery were also evaluated.

## Methods

### Inclusion and exclusion criteria

The inclusion criteria were (1) age < 55 years, (2) pain on the medial side only, (3) varus angle < 5°, (4) injury of the posterior part of the medial meniscus, (5) arthroscopic exploration showing narrowing of the medial space in the semi-straight position of knee, the posterior aspect of the medial meniscus was not clearly visible, (6) need to perform release of the PMC of the knee, and (7) postoperative follow-up time of more than 1 year. The exclusion criteria were (1) degenerative injury of the meniscus, (2) Outerbridge grade of cartilage degeneration ≥3 grade, (3) combined ligament or lateral meniscus injury, (4) previous knee operation, and (5) inflammatory arthritis. This study was approved by the Hospital Ethics Committee, and informed consents were obtained from all patients.

Based on aforementioned criteria, 60 patients with posterior injury of the medial meniscus between January 2016 and January 2018 were included in this study. The patients underwent pie-crusting release of the PMC under arthroscopy to improve the field of view of the posteromedial space of the knee. Patients comprised 32 males and 28 females with an average age of 37.0 ± 5.3 years (16–55 years). The mean body mass index (BMI) was 25.0 ± 2.1 kg/m^2^ (21.5–31.4 kg/m^2^). Thirty-eight patients had injury to the left knee and 22 to the right knee. The mean time from injury to surgery was 35.5 ± 2.1 days (7–120 days). There were 26 cases with only posterior meniscus injury, and 34 patients with posterior plus body injury. Meniscoplasty were performed in 40 patients, and suturing was performed in 20 patients. According to the O’Connor classification of meniscus injury, there was a simple horizontal tear in 20 knees, a sagittal tear in eight cases, a longitudinal tear in 20 knees, and a complex tear in 12 knees. According to the Outerbridge classification of cartilage injury, 22 cases were grade 0, 21 were gradeI, and 17 were grade II [8]. The clinical symptoms were locking, clicking, joint swelling and claudication. All operations were performed by a single surgeon.

### Surgical technique

**Fig. 1:**
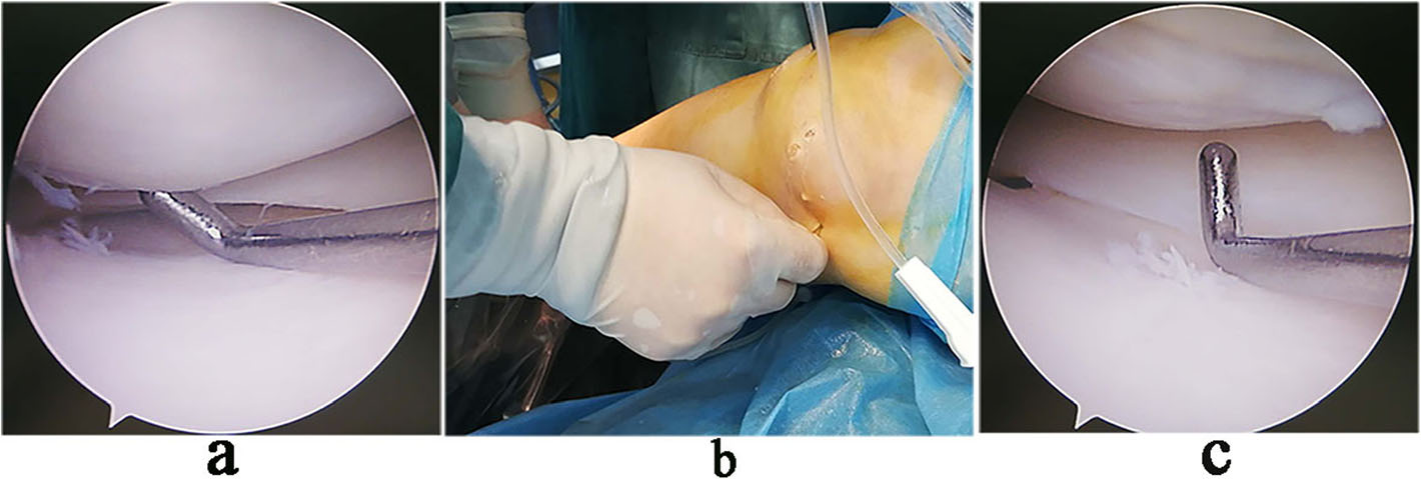
Arthroscopic pie-crusting release of the PMC. **a** With the knee joint in the valgus position, the probe did not pass vertically through the narrow medial space. **b** Multi-point pie-crusting release of the PMC with an 18-G needle. **c** After release, the probe passed vertically through the medial space, and the visual field was improved

A load cell with capacity of 50 kg and accuracy of 0.01 kg (model L6D, ZEMIC Ltd., Hanzhong, Shaanxi, China) was mounted on the lateral block post of the operating table to monitor the amount of valgus stress that was applied to the patient’s thigh during the operation. Under general or spinal anesthesia, the patient was positioned supine on the operating table with a tourniquet at the proximal thigh. Standard anterolateral (AL) and anteromedial (AM) approaches were used to examine meniscus, cartilage and ligaments. For patients with medial meniscus injury, the exposure of the posteromedial compartment was observed through the AL approach with the knee joint in a semi-straight position at 20°. Valgus and external rotation stress of 11-kg was applied to the leg by the surgeon using his body [9]. When the medial compartment was narrow, the posterior part of the medial meniscus was not clearly visible, and it was difficult to smoothly insert the blue forceps into the posterior space. Then outside-in controlled multi-point pie-crusting release of the MCL and POL were performed. The detailed procedures were as follows: (1) The probe hook was inserted into the medial joint space through AM approach, and the width of the posteromedial gap was evaluated with reference to whether the probe tip (4 mm height; Smith & Nephew plc., London, UK) could pass through the narrow medial space vertically (Fig. 1a). (2) At the near level of the medial joint line, percutaneous multi-point pie-crusting piercing of the posterior part of the MCL and the POL with an 18-G venous needle was carefully carried out after identifying the course of the saphenous nerve and vein using transillumination with the arthroscope, and it is appropriate to sense a “slight tear” when piercing the aforementioned tissues (Fig. 1b). (3) Under 11-kg valgus stress, multi-point release stopped when the posteromedial space was suddenly opened. At this time, the posterior part of the meniscus could be clearly viewed, and the probe tip could easily pass through the narrow space in the vertical orientation (Fig. 1c). (4) When the posteromedial space was opened satisfactorily, meniscoplasty or suture could be performed.

### Postoperative management

After recovery from anesthesia, isometric contraction of the quadriceps femoris and straight leg elevation training were performed. Patients who underwent meniscoplasty were recommended to wear an short knee brace for 4 weeks to prevent further injury of the MCL, and were allowed to do full-weight bearing and full range of motion exercise 2 days after operation. Patients who underwent meniscal suture were encouraged to practice ambulation without weight-bearing on the affected limbs under the protection of a adjustable brace and crutch. Four weeks after operation, patients carried out partial weight-bearing activities, then gradually the brace was removed and full weight-bearing activities were allowed 6 weeks after operation, with active knee flexion more than 120°. Stationary cycling and moderate intensity running were allowed 3 months after surgery, and full return to sport was permitted 6 months later.

### Arthroscopic measurements of the joint space width (JSW)

**Fig. 2:**
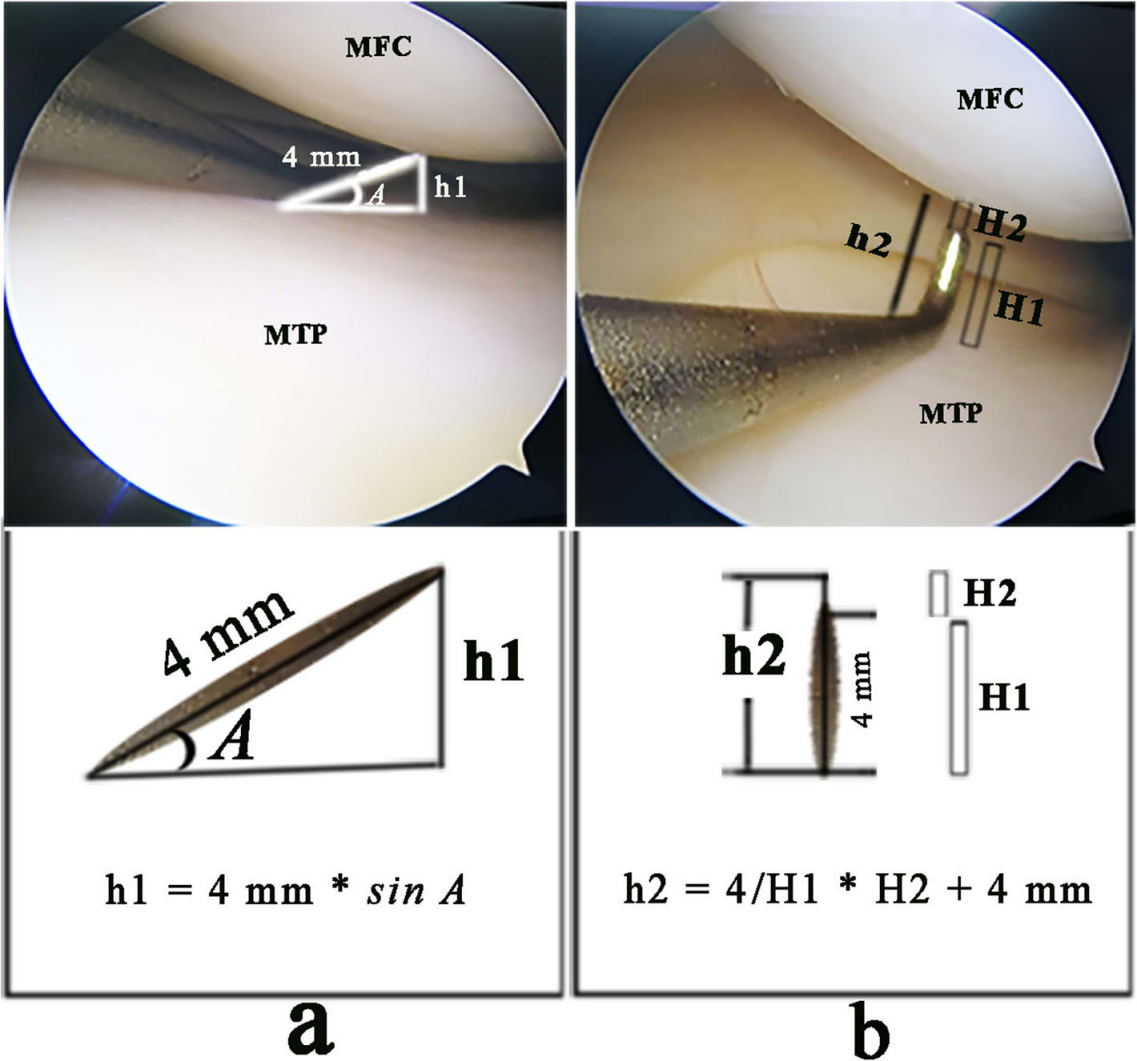
Arthroscopic measurements of the JSW. **a** Before pie-crusting release. **b** After Pie-crusting release. MFC, medial femoral condyle. MTP, medial tibial plateau

The intraoperative width of the medial space before and after release of the PMC was measured as follows. A probe hook was inserted through the AM approach, and its vertical tip was placed against the lowest point of the femoral condyle cartilage at the narrowest space. Before release, the vertical tip (4 mm) often formed an acute angle with the articular surface; The width of the narrowest space (h1) can be calculated by the sine formula (h1 = 4 mm × sin A), where A is the angle between the vertical tip and the articular surface (Fig. 2a). After release, the medial space was enlarged, and the vertical tip could pass vertically through the narrow space, and the opening width of the medial gap (h2) can be calculated by the formula (h2 = 4/H1 × H2 + 4 mm), where H1 is the magnified measured height of the vertical tip of the hook under microscopy (greater than 4 mm), and H2 is the magnified measured height between the tip of the hook and the lowest point of the femoral condyle under microscopy (Fig. 2b). h1-h2 is the extra opening width. The height of H1 and H2 was measured with a digital calliper with accuracy to 0.1 mm.

### Radiographic measurements of the JSW

**Fig. 3:**
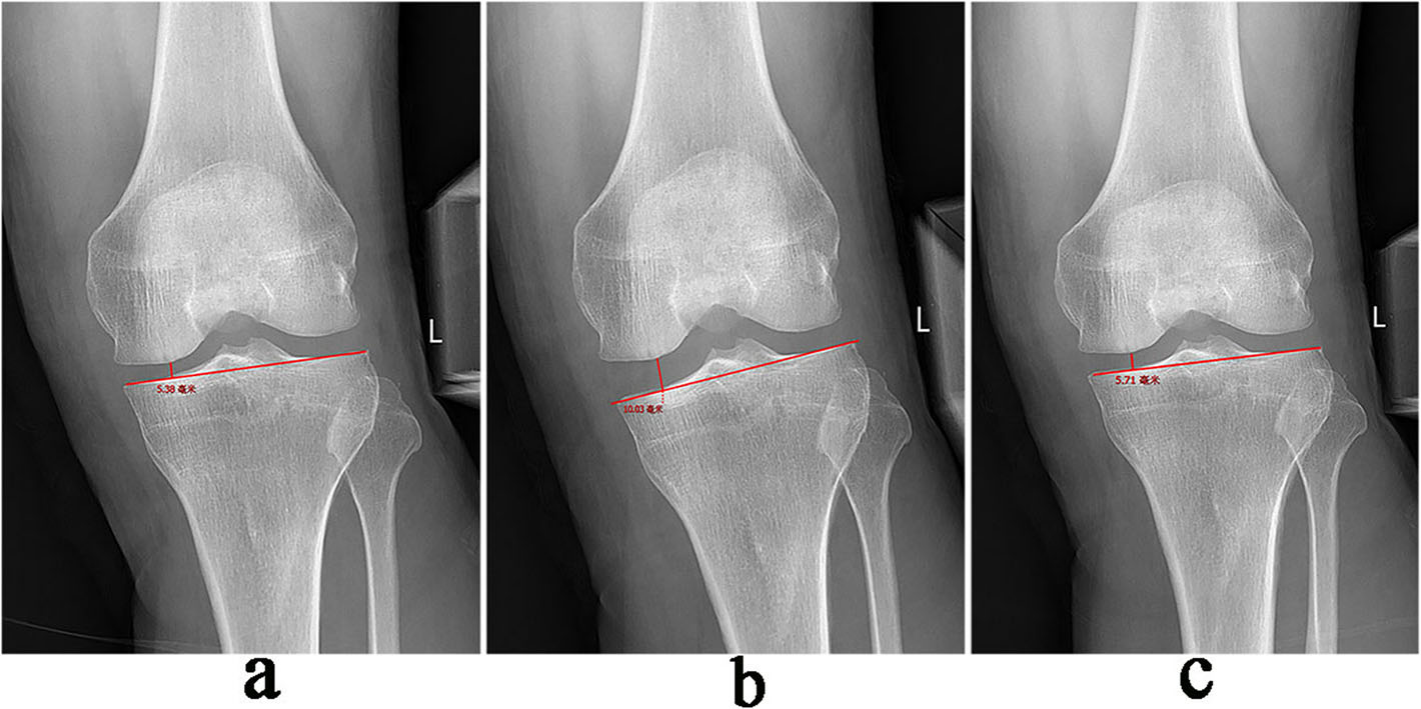
Radiographic measurement of JSW under valgus stress radiograph. The distance of JSW (perpendicular line) was measured using PACS software. **a** The JSW was 5.38 mm preoperatively. **b** 10.03 mm in the 1st week postoperatively. **c** 5.71 mm by the 3rd month postoperatively

The identical load cell was mounted on the stress radiography device to monitor the amount of valgus stress that was applied to the patient’s thigh while performing X-ray films. With the patient in supine position and the knee in a semi-straight position at 20°, an anteroposterior (AP) knee radiograph with 11-kg valgus stress of the lower leg was taken with the X-ray beam centred in the joint line and a tube distance of 1 m from the cassette. JSW was measured using a picture archiving and communication system (PACS) as follows with modification [9]. On an AP X-ray film, a line was drawn to connect the subchondral bone of the medial and lateral tibial condyle. From this line, a perpendicular line was drawn to the most distal point of the medial femoral condyle. The distance of this vertical line was recorded as JSW and measured using PACS software (PACS, PI View STAR, version 5025; Infinitt, Seoul, Korea) (Fig. 3).

### MRI analysis of healing of the meniscus and MCL

All MRI examinations of the knee was performed using a 3.0-T system before and after operation. A special superficial sagittal section was taken to evaluate the localization of the PMC injury. In coronal sections, the localization of the injury was recorded with reference to the medial meniscus [9]. Based on MRI signal grade, the meniscus was considered completely healed if there was no fluid signal within it, incompletely healed if an intrameniscal signal approached only one articular surface, and unhealed if the signal hyperintensity extended from one articular surface to the other [10].

### Clinical evaluation

To evaluate the effect of arthroscopic pie-crusting release during operation, the width of the medial space before and after release was measured; the improvement of the visual field and occurrence of iatrogenic cartilage injury were also observed. During the follow-up, the medial stability of the knee joint was evaluated by radiographic measurements of the JSW preoperatively, and at 1 week and 3 months postoperatively. Healing of the MCL and sutured meniscus was evaluated by MRI. VAS [11], Lysholm score [12], Tegner score [13], and IKDC score [14] were used to evaluate knee joint functions.

### Statistical analysis

The measurement data are expressed as mean ± standard deviation. Student’s paired t-test was used to test the significance of differences, and *P* < 0.05 denoted a significant difference. All statistical analyses were performed with SPSS 15.0 (SPSS Inc., Chicago, IL, USA).

## Results

**Fig. 4:**
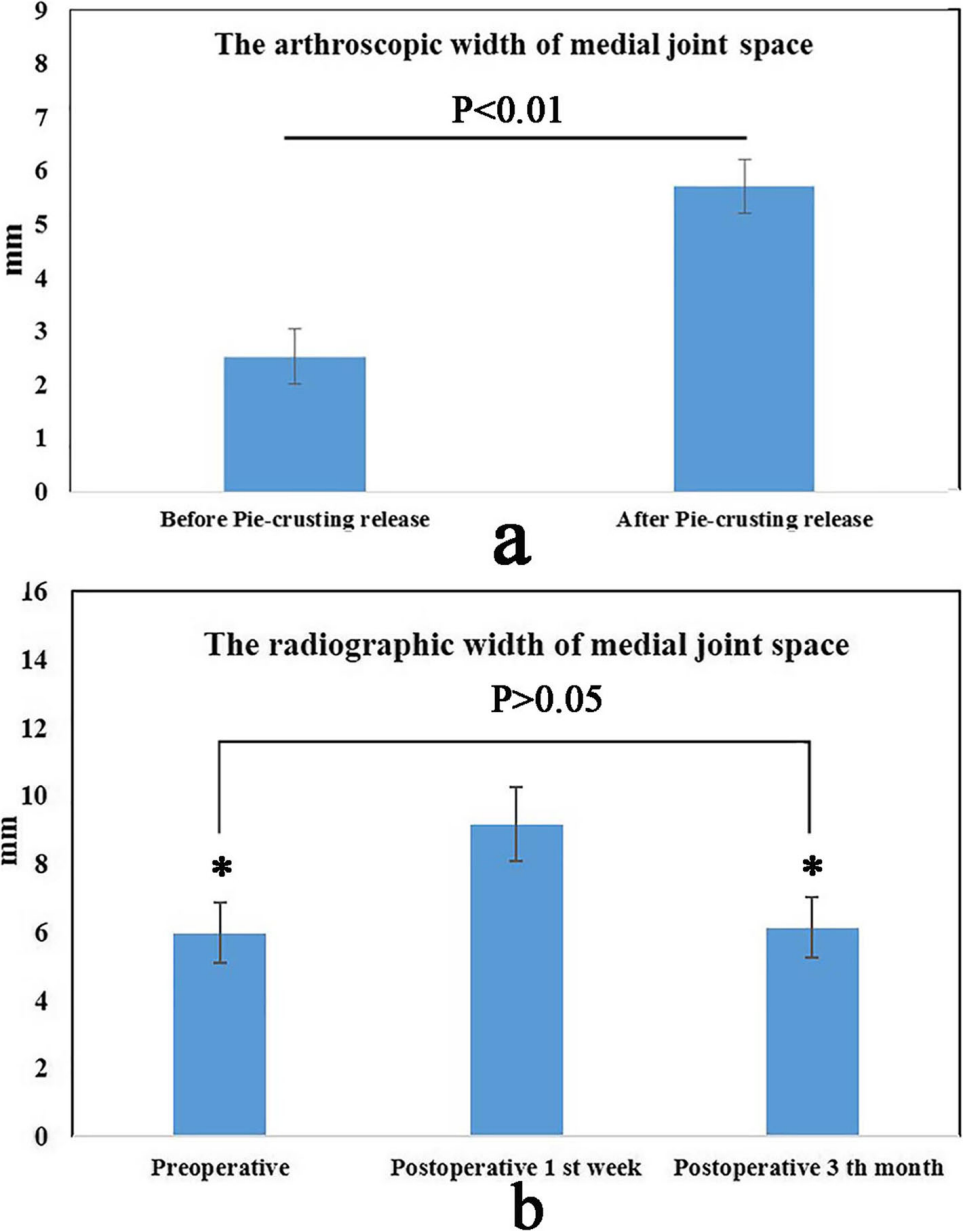
Measurement of medial JSW of the knee joint. **a** Arthroscopic measurement of the JSW. **b** Radiographic measurement of the JSW, compared with the value of 1st week postoperatively, ^*^p < 0.01

After pie-crusting release of the PMC of the knee joint, the intraoperative posteromedial gap was obviously increased. The width of the medial space, as calculated from the arthroscopic photographs and formula, was 2.5 ± 0.5 mm (1.9–4 mm) before release and 5.7 ± 0.5 mm (4–7 mm) after release, and the difference was statistically significant (*p* < 0.01) (Fig. 4a). The extra increased width was 3.2 ± 0.6 mm (2–4.4 mm). The location and morphology of the injured meniscus were clearly visible, and no obvious iatrogenic cartilage injury occurred during operation.

The patients were followed up for 21.93 ± 7.04 months (12–36 months). At the final follow-up, the JSW, as calculated from the X-ray radiographs under valgus stress, was 5.9 ± 0.8 mm (4–7.5 mm) preoperatively, 9.2 ± 1.1 mm (7–11 mm) in the 1st postoperative week, and 6.1 ± 0.9 mm (4.2–7.6 mm) by 3 months postoperatively. The difference in JSW between the preoperative measurement and that at the 1st postoperative week was statistically significant (*p* < 0.01); however, there was no significant difference between the preoperative width and that at 3 months postoperatively (*p* > 0.05) (Fig. 3, Fig. 4b).

**Fig. 5:**
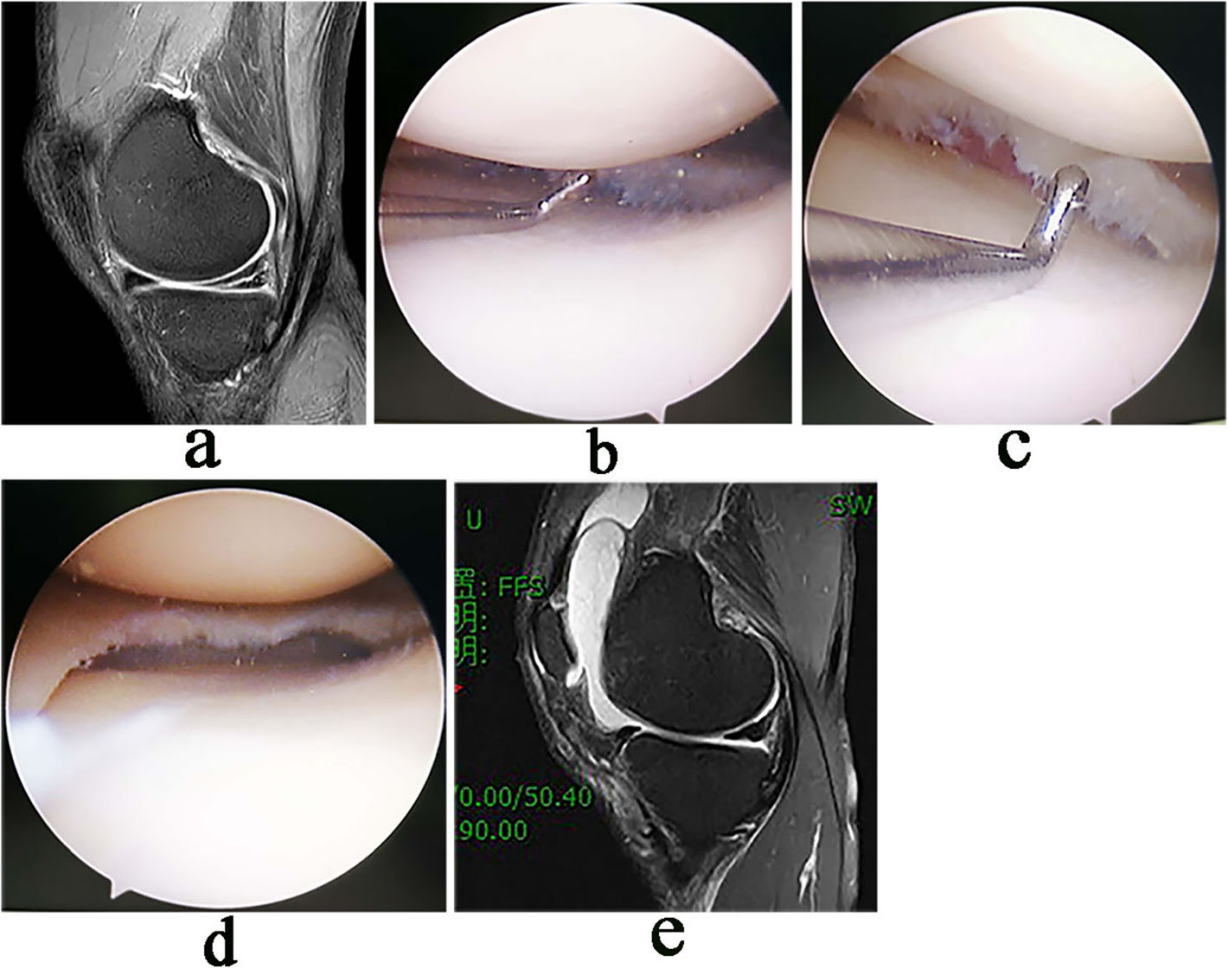
Arthroscopic pie-crusting release of PMC for meniscoplasty. **a** Preoperative MRI showed a horizontal tear of the medial meniscus. **b** Arthroscopic visual field of the posteromedial space was not clear, and the probe tip could not pass vertically through the narrow gap. **c** After release, the probe tip could pass vertically through the narrow gap, and the visual field was improved. **d** Meniscoplasty of the posterior meniscus was performed without iatrogenic cartilage injury. **e** After 18 months follow-up, the shape of the posterior meniscus was satisfactory

**Fig. 6:**
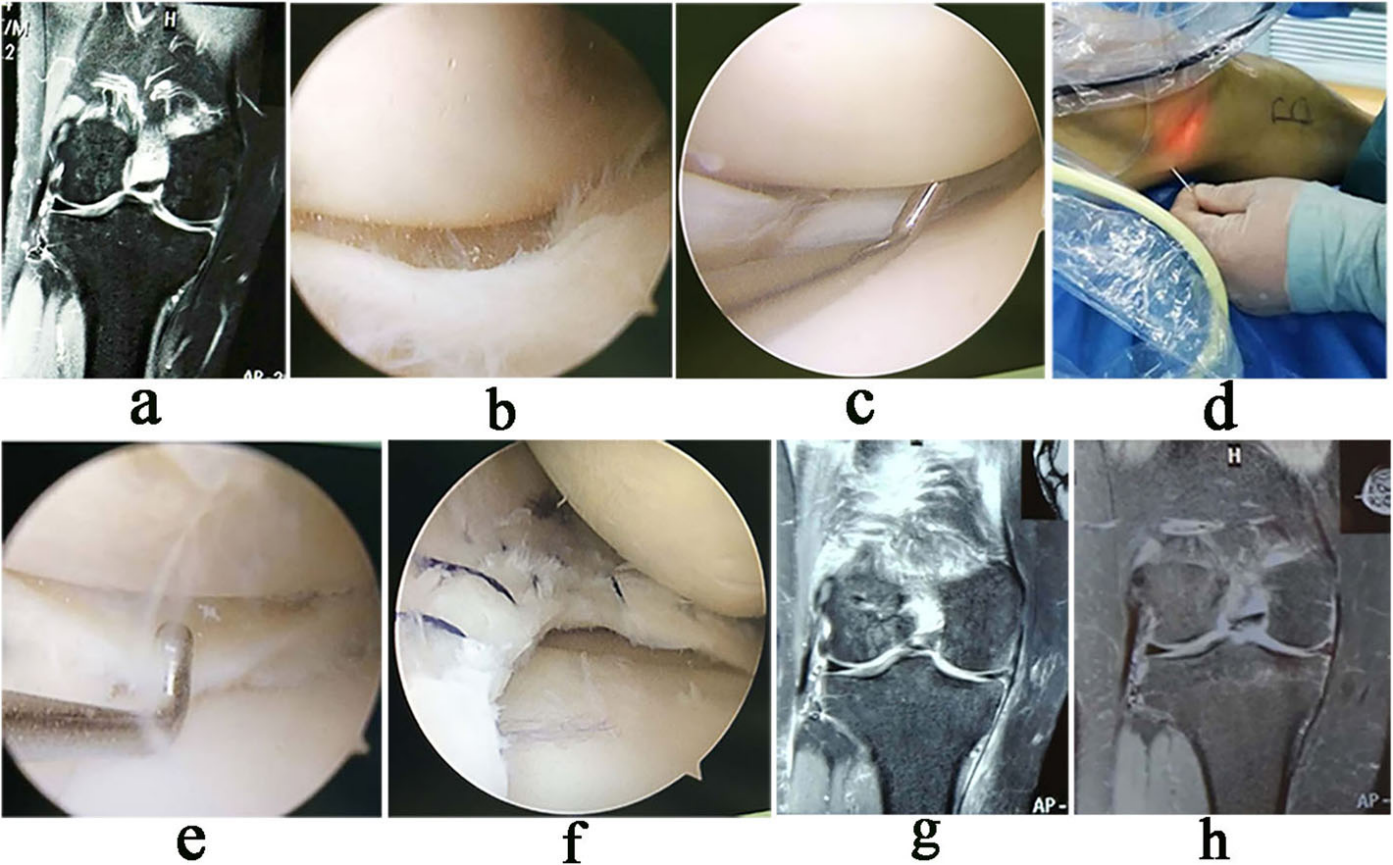
Arthroscopic pie-crusting release of PMC for meniscus suture. **a** Preoperative MRI showed a bucket-handle tear of the medial meniscus. **b** Displacement of the injured meniscus under arthroscopy. **c** The injured meniscus was reduced, but the probe tip could not pass vertically through the medial gap. **d** Multi-point release of the PMC. **e** After release, the probe tip could pass vertically through the narrow gap. **f** The meniscus was sutured, with no iatrogenic cartilage injury. **g** Postoperative MRI showed a normal meniscus shape and mild iatrogenic injury of MCL. **h** After 18 months follow-up, MRI showed healing of the meniscus and MCL

At the final follow-up, of the 20 patients with meniscus suture, MRI showed complete meniscus healing in 15 patients and two-grade abnormal signals in five patients who had no obvious tenderness in the medial space. For all patients, VAS, Lysholm, IKDC and Tegner scores were 1.80 ± 0.51 (1–3), 80.08 ± 3.74 (70–85), 82.17 ± 4.64 (75–90) and 5.48 ± 0.59 (4–7), respectively, showing significant differences compared with the preoperative scores [5.57 ± 0.69 (4–7), 48.17 ± 4.22 (40–55), 51.42 ± 4.02 (45–55) and 3.20 ± 0.68 (2–4), respectively, *p*< 0.01) (Table 1). Typical cases were shown in Fig. 5 and Fig. 6.

**Table 1:** Clinical assessment results (*n* = 60, Mean ± SD)

	Preoperation	Final follow-up	*P* value
VAS	5.57 ± 0.69	1.80 ± 0.51	0.00
Lysholm	48.17 ± 4.22	80.08 ± 3.71	0.00
IKDC	51.42 ± 4.02	82.17 ± 4.64	0.00
Tegner	3.20 ± 0.68	5.48 ± 0.59	0.00

## Discussion

For posterior injury of the medial meniscus with clinical symptoms, arthroscopic minimally-invasive surgery is often required. The difficulty during operation is that the posteromedial compartment is difficult to expose clearly because of the narrow joint gap, which results in a limited visual field of the posterior meniscus and a lack of space for manipulation of the surgical instruments under arthroscopy [2–6]. Consequently, it is important for arthroscopic surgeons to seek other methods to enlarge the posteromedial space of the knee joint, one of which is the use of the pie-crusting release technique to release the medial structure of the knee under arthroscopy in order to increase the medial joint gap, and ultimately improve the intraoperative visual field and operation space [15–17]. In total knee arthroplasty, in order to achieve the lateral and medial balance of the extension and flexion gap, Mihalko et al. performed pie-crusting acupuncture release of the MCL, and the joint space during flexion and extension was simultaneously increased and balanced [15]. Bellemans et al. used this technique to release the MCL with a 19-G needle in the vicinity of the knee joint line, which produced a mean 2.4 mm increase of the medial joint space [17]. It has been reported that application of this technique to the opening of the medial space of the knee joint under arthroscopy, whether through an open skin incision or percutaneous acupuncture for MCL release, improves the medial visual field [2, 6, 18–20]. In this study, the pie-crusting technique was used to release the PMC of the knee joint, and a satisfactory visual field of the posterior joint space was obtained under valgus stress of the knee in a half-extension position, in which not only the posterior shape of the meniscus was fully revealed, but also it was possible to insert the surgical instruments into the posterior space accurately and smoothly, and no further iatrogenic cartilage injury occurred.

To increase the width of the posteromedial space of the knee joint under arthroscopy, it is not necessary to release the whole medial stable structure. For arthroscopic treatment of medial meniscus injury in this study, the position of the lower extremity was usually near extension, in which the main structure restricting knee valgus was the posteromedial stable structure. Reports in the literature have shown that the posteromedial stable structure of the knee joint mainly consists of the posterior part of the MCL, the POL and the posteromedial capsule [21]. The anterior part of the MCL maintains tension and restricts excessive knee valgus in the flexion position. The posterior fiber of the MCL maintains tension in the extension position, restricting knee valgus in the knee half-extension position (20 degrees of flexion). The POL is the thickening part of the posteromedial side of the articular capsule, the proximal end of the POL is attached to the adductor tubercle of the femur or between the adductor tubercle and the medial femoral condyle, then expands distally and posteriorly and attaches to the posterior side of the tibia, and its main central or tibial bundle attaches to the posteromedial side of the tibial plateau near to the joint surface, preventing knee valgus in a half-extension position [21]. It can be seen from this analysis that the posterior part of the MCL and the POL are the main tissue structures which restrict the opening of the posteromedial space in the half-extension position under knee valgus. We performed percutaneous multi-point acupuncture for the posterior part of the MCL, POL and articular capsule using an 18-G needle at the posterior 1/3 region of the knee joint, and satisfactory posterior space exposure was obtained under valgus stress.

The clinical results have shown that pie-crusting release of the PMC can obviously improve the visual field of the posteromedial space of the knee joint. However, a question arising from this technique is whether the release can result in the risk of medial instability of the knee after surgery. One imaging study in the literature which reported changes in the medial gap after MCL release found that the release caused a partial tear of the MCL, and the medial space was 2 mm, 0.9 mm and 0.1 mm at 1 week, 1 month and 3 months after operation, respectively [20]. Another study showed that the MCL injury was rated grade I (< 5 mm opening on the medial side) in the valgus position with 20° flexion immediately after operation but negative at 6 weeks after operation, and there was no subjective feeling of medial instability of the knee during follow-up [6]. A similar result was also found in our study; the patients had no subjective or objective medial instability of the knee joint at 3 months after operation and the joint space width from X-ray radiographs was 6.13 ± 0.89 mm, showing no significant difference compared with preoperative 5.97 ± 0.89 mm, indicating healing of the injured PMC. The main factors related to knee stability after pie-crusting release of the PMC were as follows: (1) In this technique, only the posterior part of the MCL and POL were pierced, the anterior part of the MCL was intact. (2) The extra intraoperative opening width of the medial space after release was a mean 3.19 mm, which only maintained relaxation of the posteromedial stable structures within the range of grade I injury [9]. (3) Patients were required to wear a brace for at least 4 weeks after operation, which ensured the healing of the sutured meniscus and the released posteromedial stable structure, thus avoiding the occurrence of iatrogenic knee instability.

Currently, there are two kinds of pie-crusting release technique for the PMC during arthroscopic medial meniscus surgery according to the literature reports, that is inside-out technique and outside-in technique [2–6, 18–20]. Compared with the former, the latter used in our study has the following advantages: no invasion of the intra-articular space, easy and effective puncturing operation without risk of iatrogenic cartilage injury, controlled release of the PMC, lower hypothetical risk of infection, and needle access independent of intra-articular structures [3, 19]. On the other hand, the outside-in technique has the disadvantage of potentially injuring to the saphenous vein and nerve during the process of percutaneous PMC penetrating. However, this can be effectively avoided by familiarity with the posteromedial anatomy of the knee joint and accurate intraoperative puncture [5, 18, 19]. Another disadvantage is the over-release of the PMC, but no residual laxity described for long-time follow-up to our knowledge [6, 9, 20].

The following surgical tips for outside-in arthroscopic pie-crusting release of the PMC should be noted. (1) While performing pie-crusting release, the knee joint should be maintained in slight flexion and abduction to ensure a certain tension of the PMC [6]. (2) In order to avoid possible injury to the saphenous nerve and great saphenous vein, percutaneous puncture should be performed 1.0 cm above and below the posterior half of the joint line level, where the saphenous nerve branches and the great saphenous vein rarely passed [5]. (3) The puncture should be carried out from the posterior part to the middle part of the PMC row by row until feeling a “slight tear”. (4) The release standard should be that the probe tip could easily pass through the narrow joint space in the vertical orientation, or the blue forceps could easily enter the posterior space. (5) The knee joint should be fixed with a brace after operation to promote healing of the released MCL.

There are some limitations for this study. Firstly, conventional MRI rather than MRI arthrography was used in this study to evaluate the healing of sutured meniscus, and all pie-crusting release of PMC of the knee were performed by a single surgeon in one medical center, which might interfere with the interpretation of the reported findings. Secondly, the study is a retrospective, nonrandomized and uncontrolled design, which may be associated with the risk of patient selection and results bias. It will be convincing to embark on a high-quality prospective randomized-controlled study in which comparison groups using an alternative meniscal repair technique or non-surgical management are included. Thirdly, due to our low subject count and short follow-up period, additional patients need to be recruited with a longer follow-up time.

## Conclusions

Arthroscopic pie-crusting release of the PMC can effectively increase the posteromedial space and improve the visual field of the knee joint under arthroscopy, and this technique does not produce any residual valgus instability of the knee nor affect clinical outcome at the final follow-up.

## Data Availability

All data generated or analysed during this study are included in the supplementary information files and are available from the corresponding author on reasonable request.

## Abbreviations

MCL: Medial collateral ligament
PMC: Posteromedial complex
POL: Posterior oblique ligament
JSW: Joint space width
MRI: Magnetic resonance imaging
VAS: Visual analogue scale
IKDC: International knee documentation committee
BMI: Body mass index
AL: Anterolateral
AM: Anteromedial
